# Burden level and influencing factors of caregivers for patients with autoimmune encephalitis

**DOI:** 10.3389/fneur.2026.1763795

**Published:** 2026-07-01

**Authors:** Liyan Gu, Xinyi Han, Jili Shen, Yujun Wu, Xiaoni Liu, Jie Wei, Xiangjun Chen

**Affiliations:** 1 Department of Neurology, Huashan Hospital, Institute of Neurology, Fudan University, Shanghai, China; 2 National Center for Neurological Disorders, Shanghai, China; 3 Department of Neurology, NO. 905 Hospital of PLA Navy Affiliated to Naval Medical University, Shanghai, China; 4 Emergency Room, NO. 905 Hospital of PLA Navy Affiliated to Naval Medical University, Shanghai, China

**Keywords:** autoimmune encephalitis, burden, caregivers, cross-sectional study, influencing factors

## Abstract

**Background:**

Caregiver burden is a critical issue in neurological disorders, but remains underexplored in autoimmune encephalitis (AE), a severe condition on patients and their caregivers. Understanding the burden level and its determinants is essential for developing effective support systems. This study aimed to comprehensively assess the burden level of caregivers for patients with AE and identify key influencing factors.

**Methods:**

In a cross-sectional study, 151 AE inpatients and their primary caregivers were recruited by convenience sampling from the neurology department of a tertiary hospital in Shanghai between March 2023 and June 2024. Patient status was assessed using the Barthel Index (BI) and Modified Rankin Scale (mRS). Caregivers completed the Zarit Burden Interview (ZBI), Caregiver Preparedness Scale (CPS), and Self-Rating Anxiety Scale (SAS), alongside a sociodemographic questionnaire. Univariate, correlation, and multiple linear regression analyses were employed to identify factors associated with caregiver burden.

**Results:**

The mean ZBI score was 36.50 ± 19.97, with 24.50% of caregivers having no/mild burden, 35.76% moderate burden, and 39.74% severe burden. Multiple linear regression identified four significant predictors of caregiver burden: caregivers’ anxiety level [*β* = 0.513, 95%CI (0.381, 0.645), *p* < 0.001], ability to afford medical expenses [*β* = 0.131, 95%CI (0.010, 0.252), *p* < 0.05], previous care experience [*β* = −0.157, 95%CI (−0.270, −0.044), *p* < 0.01], and caregiver preparedness [*β* = −0.175, 95%CI (−0.301, −0.049), *p* < 0.01].

**Conclusion:**

Caregiver burden in AE is significantly associated with higher anxiety levels, greater financial strain, lack of previous care experience, and lower preparedness. Patient functional severity exerts an indirect association with caregiver burden through caregiver-related factors. These findings highlight the need for targeted interventions including mental health support, enhanced care preparation, peer mentorship initiatives, and socioeconomic assistance to reduce caregiver burden and improve care quality for AE patients.

## Introduction

Encephalitis encompasses a heterogeneous group of neurological disorders characterized by inflammatory lesions in brain parenchyma, primarily affecting gray matter and neurons while potentially involving white matter and vascular structures (1). Among its subtypes, autoimmune encephalitis (AE) represents a clinically significant category accounting for 10–20% of all encephalitis cases (2), with an estimated annual incidence of 0.8/100,000 in Western populations (3). Unlike infectious etiologies, the precise pathogenesis of AE remains incompletely understood, though it is characterized by immune-mediated neuronal dysfunction. Clinically, AE manifests predominantly with cognitive impairment or psychiatric symptoms in 60–80% of cases (4), often leading to diagnostic challenges due to its non-specific presentation (5). When inflammation involves the limbic system or spreads extensively to the entire brain, patients often develop significant abnormal mental behaviors. Such encephalitis with abnormal mental behaviors as the initial symptom, inconspicuous main symptoms, or rare types is easily misdiagnosed as other diseases, leading to delayed treatment and difficulties in disease management.

AE is associated with substantial morbidity and mortality, necessitating early intervention to improve prognosis (6, 7). Patients with severe cortical involvement often require prolonged bedridden care, resulting in functional disability and placing significant physical, emotional, and economic burdens on caregivers (8). As a chronic condition requiring long-term management, AE imposes dual challenges: not only does it incur substantial treatment costs, but it also disrupts family socioeconomic stability. Caregiver burden in neurological disorders has been extensively documented (9–11), yet specific research on AE caregivers remains limited despite their critical role in patient outcomes.

The high-intensity care demands of AE patients, including complex medical and rehabilitative needs, create unique stressors for caregivers. However, systematic investigations into the magnitude and determinants of this burden are lacking. Based on existing evidence on caregiver burden in neurological disorders and the clinical characteristics of AE, we proposed the following hypotheses: (1) Primary caregivers of AE patients experience moderate to severe levels of burden; (2) Both patient-related factors (functional severity, activities of daily living) and caregiver-related factors (anxiety level, preparedness, previous care experience, financial strain) are significantly associated with caregiver burden; (3) Caregiver-related factors have a stronger independent association with burden than patient-related factors.

This study addressed this gap by quantitatively assessing the burden levels among AE caregivers and identifying key influencing factors. By employing validated psychosocial and clinical assessment tools, we aimed to provide evidence-based insights that could inform targeted interventions to alleviate caregiver strain. Our findings contributed to the development of multidimensional support strategies for AE families, ultimately improving care quality and patient outcomes.

## Methods

### Study design and participants

This was a cross-sectional study conducted between March 2023 and June 2024 at the neurological department of a tertiary general hospital in Shanghai. The study aimed to assess the burden level of primary caregivers for patients with AE and to identify factors influencing caregiver burden. A convenience sampling approach was employed to recruit AE inpatients and their primary caregivers. The sample size was determined using G*Power software (version 3.1) with an effect size *f*^2^ of 0.15, alpha level (*α*) of 0.05, and statistical power (1-*β*) of 0.80 (calculated required sample size ≈ 129). Accounting for an estimated 20% invalid questionnaire rate, the initial recruitment target was set at 161 pairs. Ultimately, 151 valid patient-caregiver pairs were included in the analysis. The study was approved by the Institutional Review Board of the NO. 905 Hospital of the PLA Navy (approval number: 2023-Ethics Opinion-09), and all participants provided written informed consent.

*Inclusion criteria for AE patients*: ① Met the diagnostic criteria for definite or probable autoimmune encephalitis proposed by (38); ② Underwent paired serum and cerebrospinal fluid (CSF) autoimmune antibody detection, including antibodies against N-methyl-D-aspartate receptor (NMDAR), leucine-rich glioma-inactivated 1 (LGI1), contactin-associated protein-like 2 (CASPR2), gamma-aminobutyric acid B receptor (GABABR), alpha-amino-3-hydroxy-5-methyl-4-isoxazolepropionic acid receptor (AMPAR), and onconeural antibodies (Hu, Yo, Ri, Ma2, CV2/CRMP5); ③ Had a fixed caregiver.

*Inclusion criteria for caregivers*: ① Unpaid family members or close relatives of AE patients, including parents, children, spouses, and siblings (no paid caregivers were included); ② Aged 18 years or older; ③ Undertaking the care tasks for patients during hospitalization (daily living care, emotional support, and medical decision-making coordination, with medical care provided by professional nursing staff) and spending the longest care time; ④ No history of intellectual disability, dementia, or other mental illnesses; ⑤ Understanding the study content and participating voluntarily.

*Exclusion criteria for caregivers*: ① Having recent major psychological trauma; ② Suffering from severe chronic diseases, such as malignant tumors, heart/kidney failure, and respiratory failure.

*Termination criteria*: ① Death of AE patients; ② Withdrawal from the study; ③ Incomplete observation data affecting evaluation.

### Data collection

Researchers received standardized training on cross-sectional survey methods, including study protocol, inclusion/exclusion criteria, questionnaire administration, ethical requirements, and communication skills. Two trained researchers were assigned per data collection group to ensure inter-rater reliability. Eligible patient-caregiver pairs were fully informed about the study purpose, procedures, potential benefits, risks, confidentiality measures, and voluntary participation. After obtaining written informed consent, caregivers completed questionnaires through face-to-face interviews within 48 h before the patient’s hospital discharge. 159 of the 168 patient-family dyads that met the inclusion and exclusion criteria agreed to participate in the study, with a participation rate of 94.64%. Nine dyads declined to participate due to reasons such as lack of time or concerns about privacy. After the initial data collection, 151 valid dyads were retained for analysis by eliminating eight invalid ones due to significant missing value. Therefore, the final valid data utilization rate was 94.97%.

For caregivers with low education levels or difficulty understanding the questionnaire, researchers provided item-by-item explanations in a standardized, non-guided manner and completed the questionnaires on their behalf. The proportion of proxy-filled questionnaires was 5.30% (8/151). All questionnaires were completed in a quiet, private environment. Researchers immediately checked for completeness and addressed any omissions or logical inconsistencies on-site. Professional nursing and rehabilitation guidance was available from the hospital’s medical team for all caregivers during the assessment period.

Patient-related data (mRS scores, Barthel Index) were assessed by responsible nurses or attending physicians within 24–48 h of admission, following standard hospital procedures. A unified Data Collection Manual was used throughout the process, with weekly group discussions to address data collection challenges.

## Measurements

### Socio-demographic information of patients and caregivers

A self-designed questionnaire was used to collect socio-demographic information. For AE patients, the general information included sociodemographic data (gender, age, marital status, medical insurance type, education level, occupation, average household income) and disease-related data (mRS score, activities of daily living at admission). For caregivers, the general information included relationship with patients, gender, age, marital status, education level, religious belief, occupation, care duration, daily care hours, presence of chronic diseases, personality type, presence of co-caregivers, ability to afford medical expenses, disease cognition, and previous care experience.

### Caregiver assessment

Caregiver burden was assessed using the 22-item Chinese version of the Zarit Burden Interview (ZBI). The original ZBI is a widely used self-report scale developed by Zarit et al. (12). The Chinese version was translated and validated by Lu et al. (13), which retained all 22 items from the original scale. Through exploratory factor analysis, Lu et al. (13) extracted two core dimensions consistent with the original scale: personal burden (12 items) and responsibility burden (7 items). Two items were not included in either dimension due to factor loadings <0.4 in the Chinese population and item 22 is an overall burden assessment item. Each item is scored on a 5-point Likert scale ranging from 0 (“never”) to 4 (“almost always”). The total score of the full 22-item scale ranges from 0 to 88, with higher scores indicating a heavier caregiver burden. Burden severity is classified using the internationally accepted standard: 0–19 points = no or mild burden, 20–39 points = moderate burden, and ≥40 points = severe burden. The Chinese version demonstrated excellent internal consistency (Cronbach’s *α* = 0.87).

Caregiver preparedness was evaluated using the Caregiver Preparedness Scale (CPS), developed by Archbold et al. (14) and validated in Chinese by Liu Yanjin et al. (15), with a Cronbach’s *α* coefficient of 0.925 and a test–retest reliability of 0.807. The scale measures caregivers’ perceived readiness across eight items: addressing patients’ physical needs, addressing patients’ emotional needs, identifying and arranging necessary services for patients, managing caregiving-related stress, providing mutually satisfying care, handling medical emergencies, accessing help and information from the healthcare system, and overall caregiving preparedness. A 5-point Likert scale was used (0 = “not at all prepared” to 4 = “very well prepared”), with a total score of 32. Higher scores indicate greater preparedness.

The Self-Rating Anxiety Scale (SAS), compiled by Zung and translated into Chinese by Wang Zhengyu (16), was used to assess caregiver anxiety symptoms, with a Cronbach’s *α* coefficient of 0.759. A 4-point Likert scale was used, with reverse scoring for items 5, 9, 13, 17, and 19. The standard score is calculated by summing the scores of 20 items and multiplying by 1.25. According to the Chinese norm, standard score <50 indicates no anxiety, 50–59 mild anxiety, 60–69 moderate anxiety, and ≥70 severe anxiety. Lower scores indicate milder anxiety symptoms.

### Patient assessment

Patient-related data included Modified Rankin Scale (mRS) scores and activities of daily living at admission, assessed using the Barthel Index. The mRS is a global disability scale measuring patients’ independence, with scores ranging from 0 (no symptoms) to 6 (death). Clinically, mRS ≤ 2 is considered indicative of good prognosis (test–retest reliability: Kappa = 0.81–0.95) (17, 18). The Barthel Index evaluates activities of daily living across 10 dimensions, with a total score of 100 (higher scores indicate greater independence; Cronbach’s *α* = 0.916; test–retest reliability = 0.854) (19).

### Statistical analysis

Data were entered into SPSS 27.0 by two independent researchers with cross-checking to ensure accuracy. Categorical variables were presented as counts and percentages. Continuous variables were expressed as means ± standard deviations if normally distributed, or as medians (interquartile ranges) if not. Normality was assessed using the Shapiro–Wilk test.

For univariate analysis, independent samples *t*-tests and one-way Analysis of Variance (ANOVA) were used for normally distributed continuous variables, while Spearman correlation analysis was applied for non-normally distributed continuous variables. The Kruskal–Wallis H test was used for comparisons between groups. Minimally adjusted univariate linear regression models (adjusted for caregiver age and sex) were additionally performed for all predictors to provide more robust univariate associations.

Multiple linear regression analysis was conducted to identify factors influencing caregiver burden, with ZBI total score as the dependent variable. Covariates were selected based on *a priori* clinical relevance, literature-driven evidence, and statistical significance (*p* < 0.05) in univariate analysis. The final model included caregiver anxiety level, caregiver preparedness, previous care experience, ability to afford medical expenses, and patient mRS score. The assumptions of normality, linearity, and homogeneity of variance were confirmed for the regression analysis. A sensitivity analysis was performed by including all statistically significant variables from Table 1 (patient monthly household income, occupation, mRS grade, activities of daily living at admission) into the model to evaluate the robustness of the main associations. Mediation analysis was conducted to explore the indirect association of patient mRS score on caregiver burden. Multicollinearity between predictors was assessed using the Variance Inflation Factor (VIF), with VIF < 2 indicating no significant multicollinearity. Residual diagnostics were performed to verify the assumptions of linear regression: the Shapiro–Wilk test was used to confirm residual normality, and the Breusch–Pagan test was used to confirm homoscedasticity of residuals. Residual-versus-fitted and normal Q–Q plots were visually inspected to further validate model assumptions. Common method variance was assessed using Harman’s single-factor test, with the first unrotated factor explaining <40% of total variance considered as no severe common method bias. Statistical significance was set at *p* < 0.05.

**Table 1:** Socio-demographic characteristics of patients, comparing ZBI, *N* = 151.

Variables	Number	Proportion (%)	ZBI score (mean ± SD)	*p* value
Medical insurance type				0.521
Medical insurance	103	68.21	37.49 ± 20.95	
New rural cooperative medical care	29	19.21	33.83 ± 12.97	
Self-payment	19	12.58	35.26 ± 23.59	
Age (years)				0.114
≤25	43	28.48	41.23 ± 22.82	
26–40	32	21.19	30.94 ± 17.82	
41–55	35	23.18	33.89 ± 20.44	
56–70	26	17.22	35.08 ± 15.50	
≥71	15	9.93	43.40 ± 18.82	
Gender				0.223
Male	76	50.33	38.47 ± 20.15	
Female	75	49.67	34.51 ± 19.72	
Monthly household income (RMB)				0.001**
<3,000	35	23.18	46.03 ± 20.52	
3,000–5,000	49	32.45	35.10 ± 17.82	
5,000–8,000	32	21.19	37.78 ± 19.93	
>8,000	35	23.18	27.77 ± 18.77	
Education level				0.201
Primary school or below	27	17.88	39.33 ± 18.71	
Junior high school	35	23.18	39.23 ± 21.31	
Senior high/technical secondary school	47	31.13	37.81 ± 19.39	
College or above	42	27.81	30.95 ± 19.85	
Occupation				<0.001***
Employed	52	34.44	28.77 ± 18.00	
Unemployed/retired	99	65.56	40.57 ± 19.84	
mRS grade				0.002**
No symptoms	6	3.97	17.00 ± 14.74	
Symptoms present but no impairment	49	32.45	29.84 ± 18.19	
Mild disability	22	14.57	36.18 ± 17.47	
Moderate disability	19	12.58	40.11 ± 22.54	
Severe disability	27	17.88	44.22 ± 19.03	
Extremely severe disability	28	18.54	42.71 ± 19.72	
Activities of daily living at admission (BI)				<0.001***
No dependence	42	27.81	27.29 ± 17.20	
Mild dependence	59	39.07	35.64 ± 20.43	
Moderate dependence	9	5.96	46.22 ± 18.32	
Severe dependence	8	5.30	45.75 ± 21.54	
Complete disability	33	21.85	44.88 ± 17.77	

**p < 0.01; ***p < 0.001. SD = standard deviation.

## Results

### Participant characteristics

Among the 151 patients, 76 were male (50.33%) with a mean age of 43.59 ± 18.98 years, and 75 were female (49.67%) with a mean age of 39.15 ± 19.05 years. The majority of patients had senior high school (technical secondary school) education (31.13%), held medical insurance (68.21%), and were unemployed or retired (65.56%; Table 1). The median mRS score was 3 [interquartile range (IQR) 2–4], and the mean Barthel Index score was 64.47 ± 37.06, indicating a wide range of functional dependence among patients. Patient mRS score was positively correlated with caregiver ZBI score (*r* = 0.312, *p* < 0.001) in univariate analysis.

Among the 151 caregivers, the average age was 46.84 ± 11.34 years: 65 males with an average age of 44.37 ± 12.03 years, and 86 females with an average age of 48.71 ± 10.47 years. Most caregivers had a junior high school education level (36.42%), no religious belief (79.47%), and were employed (49.67%). The majority of caregivers were spouses (39.07%, *n* = 59) or parents (38.41%, *n* = 58) of the patients (Table 2). Caregiver anxiety (SAS score) averaged 46.61 ± 10.71, preparedness (CPS score) averaged 17.56 ± 8.59. Subgroups with *n* < 5 are marked with † in Table 2, and their results should be interpreted with caution.

**Table 2:** Socio-demographic characteristics of caregivers, comparing ZBI, *N* = 151.

Variables	Number	Proportion (%)	ZBI score (mean ± SD)	*p* value
Relationship with patient				0.826
Spouse	59	39.07	35.32 ± 18.63	
Child	28	18.54	35.21 ± 17.90	
Parent	58	38.41	37.93 ± 22.73	
Sibling	6	3.97	40.33 ± 15.97	
Gender				0.414
Male	65	43.05	34.97 ± 19.60	
Female	86	56.95	37.66 ± 20.29	
Age (years)				0.722
≤25^†^	1	0.66	11.00 ± 0.00	
26–40	43	28.48	37.95 ± 19.11	
41–55	80	52.98	36.21 ± 20.29	
56–70	23	15.23	35.26 ± 21.71	
≥71^†^	4	2.65	40.25 ± 15.90	
Marital status				0.193
Unmarried	8	5.30	30.50 ± 20.94	
Married/cohabiting	137	90.73	36.26 ± 19.63	
Divorced/separated	5	3.31	46.20 ± 24.75	
Widowed^†^	1	0.66	70.00 ± 0.00	
Education level				0.261
Primary school or below	11	7.28	37.36 ± 24.33	
Junior high school	55	36.42	35.82 ± 19.87	
Senior high/technical secondary school	50	33.11	40.42 ± 20.81	
College/bachelor or above	35	23.18	31.71 ± 16.95	
Religious belief				0.051
No	120	79.47	34.89 ± 18.94	
Yes	31	20.53	42.74 ± 22.84	
Current work status				0.649
Employed	75	49.67	35.68 ± 19.10	
Unemployed	15	9.93	40.47 ± 20.95	
Jobless	23	15.23	40.09 ± 22.14	
Retired	32	21.19	35.50 ± 21.31	
Others	6	3.97	28.50 ± 12.82	
Care duration (days)				0.382
<30	82	54.30	35.50 ± 20.31	
30–100	33	21.85	34.12 ± 16.44	
100–200	19	12.58	43.42 ± 22.88	
>200	17	11.26	38.24 ± 21.09	
Daily care hours				0.170
1–6	25	16.56	32.92 ± 23.44	
7–12	60	39.74	40.63 ± 20.79	
13–18^†^	4	2.65	42.25 ± 21.69	
19–24	62	41.06	33.58 ± 17.04	
Presence of chronic diseases				0.913
No	122	80.79	36.59 ± 20.47	
Yes	29	19.21	36.14 ± 18.06	
Personality type				0.772
Introverted	31	20.53	37.16 ± 21.01	
Extroverted	49	32.45	37.86 ± 20.60	
Neutral	71	47.02	35.28 ± 19.27	
Presence of co-caregivers				0.664
Yes	71	47.02	35.76 ± 17.25	
No	80	52.98	37.16 ± 22.20	
Ability to afford medical expenses				<0.001***
No difficulty	26	17.22	26.81 ± 17.55	
Barely manageable	62	41.06	32.34 ± 17.25	
Some difficulty	63	41.72	44.60 ± 20.61	
Disease cognition				<0.001***
No understanding	55	36.42	45.95 ± 22.35	
Partial understanding	84	55.63	32.50 ± 16.28	
Comprehensive understanding	12	7.95	21.25 ± 12.88	
Previous care experience				0.014*
No	92	60.93	39.70 ± 19.98	
Yes	59	39.07	31.53 ± 19.08	
Anxiety level				<0.001***
No anxiety	96	63.58	27.55 ± 15.37	
Mild anxiety	33	21.85	44.33 ± 15.01	
Moderate anxiety	18	11.92	62.17 ± 13.87	
Severe anxiety^†^	4	2.65	71.25 ± 13.02	

*p < 0.05; ***p < 0.001; ^†^ Subgroup with *n* < 5, results should be interpreted with caution due to limited statistical power.

### Caregiver burden levels

The mean ZBI total score was 36.50 ± 19.97. The personal burden dimension averaged 18.50 ± 12.14, while the responsibility burden dimension averaged 11.68 ± 6.58. According to established ZBI criteria, 60 caregivers (39.74%) reported severe burden (ZBI ≥ 40), 54 (35.76%) reported moderate burden (ZBI 21–39), and 37 (24.50%) reported no/mild burden (ZBI ≤ 20; Table 3).

**Table 3:** Current status of caregiver burden, *N* = 151.

Burden level	*N* (%)	Personal burden (mean ± SD)	Responsibility burden (mean ± SD)	ZBI total score (mean ± SD)	*p* value	Post-hoc comparison
No/mild burden	37 (24.50)	5.73 ± 3.48	4.54 ± 2.65	13.84 ± 4.90	<0.001***	1 vs. 2**, 1 vs. 3***
Moderate burden	54 (35.76)	13.56 ± 4.33	9.44 ± 3.15	28.69 ± 5.58	<0.001***	2 vs. 3***
Severe burden	60 (39.74)	30.83 ± 8.44	18.10 ± 4.29	57.52 ± 12.01	<0.001***	–
Total	151 (100)	18.50 ± 12.14	11.68 ± 6.58	36.50 ± 19.97	–	–

**p < 0.01; ***p < 0.001. Post-hoc comparisons were performed with Bonferroni correction. 1 = No/mild burden; 2 = Moderate burden; 3 = Severe burden.

### Factors associated with caregiver burden

**Figure 1:**
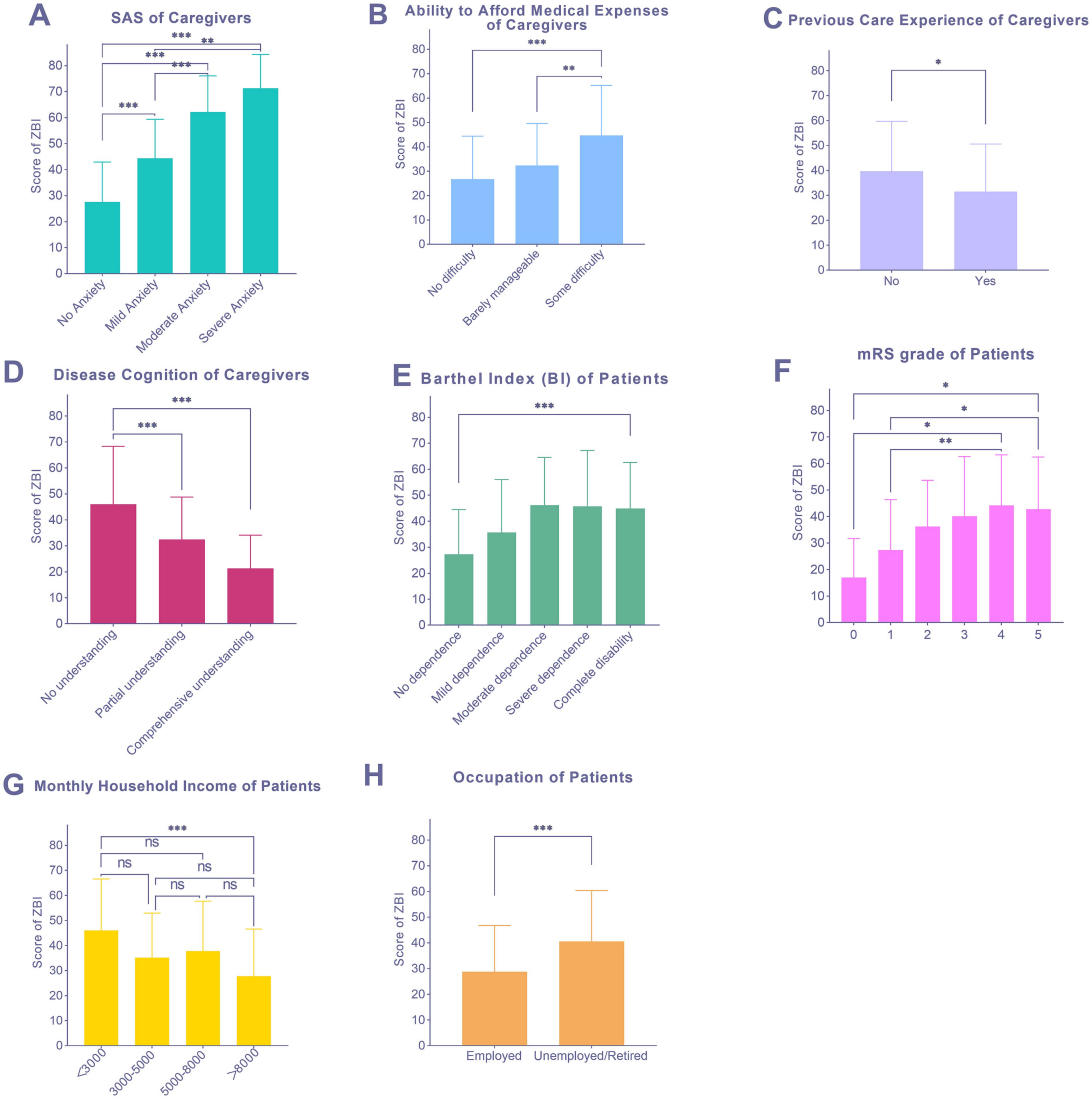
Univariate analysis of caregiver burden across different variables. Subfigures **(A)–(H)** showed univariate analysis of caregiver burden across different variables, with subfigures ranked by the absolute value of the standardized coefficient (*β*) of each predictor. Only statistically significant variables are presented (*n* = 151). ns = no statistical significance; *p < 0.05; **p < 0.01; ***p < 0.001. The four core predictors identified in the multivariable model are highlighted with distinct colors in subfigures **(A)–(D)**.

Independent samples *t*-tests were used for two-group comparisons, and one-way ANOVA or Kruskal–Wallis H tests were used for multi-group comparisons. The results (presented in Table 1 and Figure 1) showed that caregiver burden scores differed significantly by patient monthly household income (*p* = 0.001), occupation status (*p* < 0.001), mRS grade (*p* = 0.002), and activities of daily living at admission (*p* < 0.001). For caregivers, significant differences were observed by ability to afford medical expenses (*p* < 0.001), disease cognition level (*p* < 0.001), previous care experience (*p* = 0.014), and anxiety level (*p* < 0.001).

**Figure 2:**
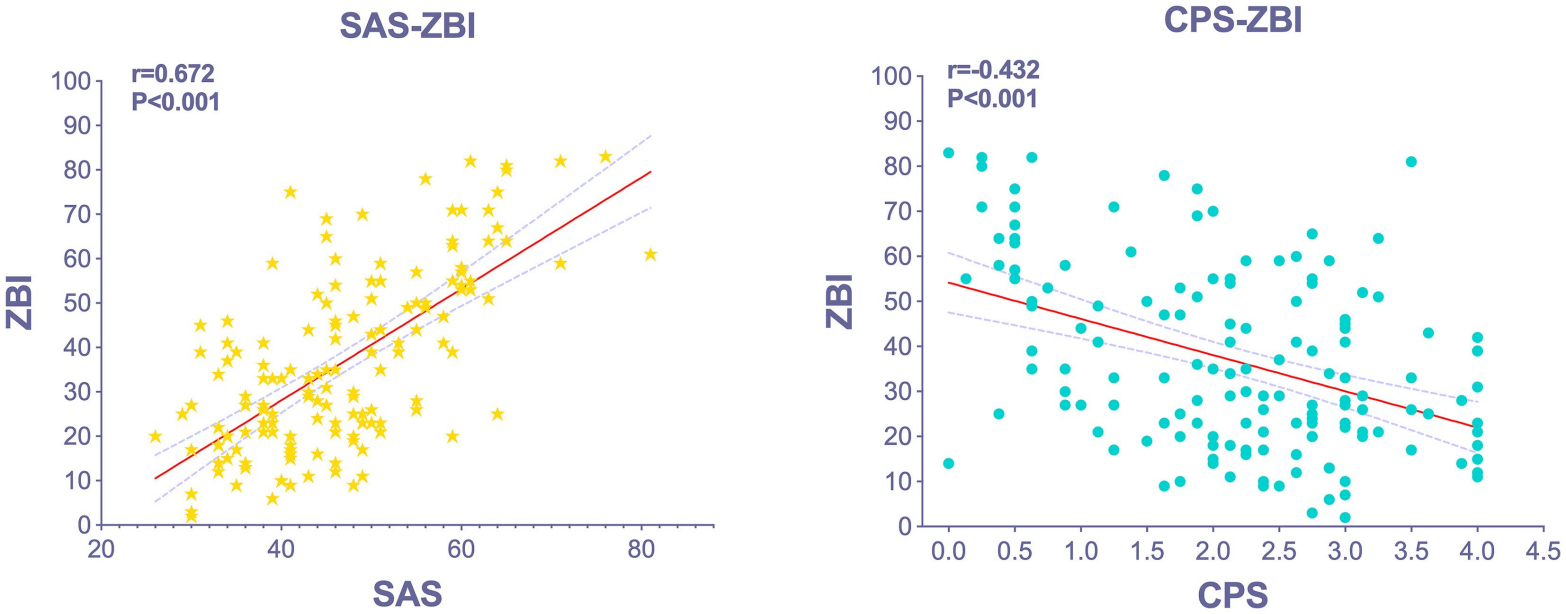
Pearson correlation analysis of caregiver anxiety level (SAS), caregiver preparedness (CPS), and caregiver burden (ZBI); *r* = −0.432 (CPS vs. ZBI), *p* < 0.001; *r* = 0.672 (SAS vs. ZBI), *p* < 0.001.

Pearson correlation was used for normally distributed continuous variables, and Spearman correlation was used for non-normally distributed variables. Caregiver anxiety level (SAS score) was positively correlated with burden (*r* = 0.672, *p* < 0.001), while caregiver preparedness (CPS score) was negatively correlated with burden (*r* = −0.432, *p* < 0.001; Table 4, Figure 2).

**Table 4:** Correlation analysis of caregiver preparedness, anxiety level, and burden, *N* = 151.

Variable	Caregiver burden score (ZBI)	Caregiver preparedness score (CPS)	Caregiver anxiety score (SAS)
Caregiver burden score (ZBI)	–	−0.432**	0.672**
Caregiver preparedness score (CPS)	−0.432**	–	−0.455**
Caregiver anxiety score (SAS)	0.672**	−0.455**	–

**p < 0.01.

### Multivariate analysis of caregiver burden

Multiple linear regression analysis was conducted to identify independent predictors of caregiver burden, with ZBI total score as the dependent variable. The final model included caregiver anxiety level, caregiver preparedness, previous care experience, ability to afford medical expenses, and patient mRS score. The assumptions of normality, linearity, and homogeneity of variance were confirmed, and no significant multicollinearity was observed (all VIF values ranged from 1.012 to 1.486, all <1.5, detailed in Table 5).

**Table 5:** Multivariable linear regression analysis with ZBI as the dependent variable (*N* = 151).

Variable	Standardized coefficient (*β*)	Variance inflation factor (VIF)	*t* value	*p* value	95% confidence interval
Constant	–	–	−1.454	0.148	(−0.114, 0.114)
Patient mRS score	0.104	1.486	1.706	0.090	(−0.016, 0.224)
Ability to afford medical expenses	0.131	1.087	2.125	0.035*	(0.010, 0.252)
Previous care experience	−0.157	1.012	−2.729	0.007**	(−0.270, −0.044)
Caregiver preparedness (CPS)	−0.175	1.243	−2.731	0.007**	(−0.301, −0.049)
Caregiver anxiety level (SAS)	0.513	1.321	7.549	<0.001***	(0.381, 0.645)

Variable coding	
Independent variable	Coding method
Patient-related factors	
Monthly household income (RMB)	<3,000 (reference, coded 1), 3,000–5,000 (coded 2), 5,000–8,000 (coded 3), >8,000 (coded 4)
Patient occupation	Unemployed/retired (reference, coded 0), Employed (coded 1)
mRS grade	No symptoms (reference, coded 0) to Severe disability (coded 5)
Activities of daily living at admission	Entered as raw value (per 1-point increase)
Caregiver-related factors	
Previous care experience	No (reference, coded 0), Yes (coded 1)
Ability to afford medical expenses	No difficulty (reference, coded 1), Barely manageable (coded 2), Some difficulty (coded 3)
Disease cognition	No understanding = 1, Partial understanding = 2, Comprehensive understanding = 3, Complete understanding = 4
Caregiver anxiety level (SAS)	Entered as raw value (per 1-point increase)
Caregiver preparedness (CPS)	Entered as raw value (per 1-point increase)

*R*^2^ = 0.531, adjusted *R*^2^ = 0.515; All VIF values < 1.5 indicate no significant multicollinearity; *p < 0.05; **p < 0.01; ***p < 0.001.

After adjustment, the analysis identified four significant predictors of caregiver burden: caregivers’ anxiety level [*β* = 0.513, 95%CI (0.381, 0.645), *p* < 0.001], ability to afford medical expenses [*β* = 0.131, 95%CI (0.010, 0.252), *p* < 0.05], previous care experience [*β* = −0.157, 95%CI (−0.270, −0.044), *p* < 0.01], and caregiver preparedness [*β* = −0.175, 95%CI (−0.301, −0.049), *p* < 0.01]. Patient mRS score showed a marginally non-significant independent association in the final model [*β* = 0.104, 95%CI (−0.016, 0.224), *p* = 0.090]. The final regression model explained 53.1% of the variance in caregiver burden (*R*^2^ = 0.531, adjusted *R*^2^ = 0.515, *p* < 0.001).

Sensitivity analysis including all statistically significant variables from Table 1 (patient monthly household income, occupation, mRS grade, activities of daily living at admission) showed that the four core predictors remained statistically significant, with consistent direction and similar magnitude of effect sizes, confirming the robustness of our main findings. Mediation analysis revealed that patient mRS score exerted a significant indirect association with caregiver burden through caregiver anxiety level and preparedness [*β* = 0.218, 95%CI (0.102–0.356), *p* < 0.001].

All VIF values were <1.5 (range: 1.012–1.486), indicating no significant multicollinearity between predictors. Notably, the VIF for caregiver anxiety level was 1.321, well below the 2.0 threshold, confirming no problematic collinearity between anxiety and caregiver burden despite their conceptual similarity. Residuals were normally distributed (Shapiro–Wilk test, *p* = 0.231) and homoscedastic (Breusch–Pagan test, *p* = 0.315). Visual inspection of residual-versus-fitted and normal Q–Q plots showed no systematic deviations from model assumptions. Harman’s single-factor test showed that the first unrotated factor explained only 31.8% of the total variance, well below the 40% threshold, indicating no severe common method bias in the study.

## Discussion

This study assessed caregiver burden among primary caregivers of patients with AE and identified key factors influencing this burden. The mean ZBI score of 36.50 ± 19.97 indicates that caregivers of AE patients experience substantial burden, with 75.5% reporting moderate-to-severe burden (ZBI ≥ 21). This burden level is higher than that reported for caregivers of patients with mild to moderate dementia or stroke (20–22), but comparable to caregivers of patients with severe dementia (23, 24). The acute onset, unpredictable course, and high medical complexity of AE likely contribute to this elevated burden, as caregivers often face unexpected challenges without adequate preparation.

The burden of primary caregivers of AE patients is influenced by multiple interrelated factors. This study initially identified potential influencing factors through univariate analysis, where patients’ monthly household income, occupation status, mRS grade, activities of daily living at admission, as well as caregivers’ ability to afford medical expenses, disease cognition level, previous care experience, and anxiety level showed statistically significant differences in caregiver burden scores. However, multiple linear regression analysis adjusted for confounding factors revealed that only four caregiver-related factors (anxiety level, preparedness, previous care experience, and ability to afford medical expenses) exerted independent and statistically significant effects on caregiver burden (*p* < 0.05; Table 5). Patient functional severity (mRS score) did not show an independent direct association, but exerted a significant indirect association with caregiver burden through caregiver anxiety and preparedness. The non-significance of patient-related factors in the multivariate model may be attributed to the mediating or confounding roles of caregiver-specific characteristics, suggesting that caregiver-centric factors are more critical in determining care burden among AE patient caregivers.

Consistent with the findings of Cejalvo et al. (25), caregivers’ anxiety level emerged as a key determinant of care burden. This association has critical clinical relevance: reducing caregiver anxiety could be the most effective target for intervention to alleviate burden. Caregivers with high anxiety tend to excessively worry about the patient’s disease progression, prognosis, and potential care challenges. This persistent emotional arousal amplifies the perceived difficulty of daily care tasks, leading to a significant increase in subjective burden. Conversely, long-term care pressure can induce sleep disturbances and social isolation among caregivers, further exacerbating anxiety as supported by Ershad Ahmad Khan et al. (26), who noted that care burden severity is positively correlated with anxiety levels (27). Such anxiety not only impairs caregivers’ mental health but also interferes with their ability to make rational medical decisions, potentially exerting adverse effects on both caregivers’ and patients’ physical and mental wellbeing (28, 29).

Caregiver preparedness, encompassing psychological readiness, mastery of care skills, and understanding of disease management, was identified as a significant protective factor against care burden. Caregivers with adequate preparedness are more capable of anticipating care needs, coping with unexpected situations, and minimizing feelings of helplessness. In contrast, insufficient preparedness may lead to feelings of inadequacy and overwhelm when facing care challenges, thereby increasing burden. This aligns with the logical framework that proactive preparation enhances caregivers’ sense of control, reducing the psychological and physical strain associated with caregiving. Although not extensively elaborated in prior studies on AE caregivers, this finding echoes broader research indicating that care preparation interventions effectively alleviate burden in other chronic disease contexts (30).

Previous care experience significantly influences caregivers’ burden levels. Caregivers with prior caregiving experience tend to possess more practical skills, better emotional resilience, and a more realistic understanding of care demands (31). This experience enables them to streamline care processes, manage stress more effectively, and adapt to the caregiving role with greater ease, resulting in lower perceived burden (32). In contrast, first-time caregivers often face a steep learning curve, struggling with unfamiliar care tasks and emotional adjustments, which amplifies their burden. This finding underscores the value of leveraging experienced caregivers as peer supporters to facilitate role adaptation for novice caregivers.

Economic capacity, specifically the ability to afford medical expenses, emerged as a critical socioeconomic determinant of care burden. Caregivers who face financial strain due to high medical costs, medication fees, and rehabilitation expenses often experience increased anxiety and psychological pressure. The persistent worry about financial sustainability diverts cognitive resources from care tasks, exacerbates emotional exhaustion, and reduces the ability to access supportive resources. In contrast, caregivers with adequate financial resources can alleviate care burden by delegating care responsibilities and accessing high-quality medical and supportive services. This aligns with the broader literature (33, 34) linking economic stress to increased caregiving burden across various disease populations.

The concept of caregiver preparedness was first proposed in 1990 (14). It refers to the perceived level of family members when assuming the caregiver role, including their ability and preparedness to meet patients’ physical, psychological, and emotional needs. Caregiver preparedness is negatively correlated with care burden: lower preparedness is associated with higher burden scores and heavier burden, which is consistent with previous studies (35). Caregivers who are inadequately prepared in terms of care knowledge, skills, and psychological coping are more likely to feel overwhelmed and out of control when facing complex care needs, leading to increased burden. Archbold et al. (14) pointed out that improving caregiver preparedness can effectively reduce their role strain, thereby lowering the burden level. Caregivers with a good understanding of the disease, mastery of care skills, and effective coping strategies have higher preparedness; such caregivers report significantly lower burden than those with low preparedness when facing the same care needs.

AE often has an acute onset, which gives caregivers very little time to respond and prepare, potentially leading to insufficient care preparedness. Due to the rapid progression of the disease, caregivers may not have fully adjusted their mindset or mastered the necessary care knowledge and skills, resulting in inadequate preparation when coping with patients’ needs. Novice caregivers often lack experience and are prone to acute anxiety when patients experience sudden conditions, leading to a strong sense of subjective burden. Sustained care may lead to physical exhaustion and psychological exhaustion. However, some caregivers may gradually adapt to the role through long-term practical experience, improving their care ability and reducing the burden level (36).

Ganapathy et al. (37) pointed out that the family economic loss caused by caregivers’ work absence further increases the care burden; thus, economic income is an important factor affecting care burden, which is consistent with the results of this study. Due to the intractability of AE, the treatment cost is relatively high, which may lead to family economic difficulties. Especially when medical insurance reimbursement is insufficient or self-payment is required, caregivers may interrupt their work due to economic pressure, further exacerbating income loss. Increased economic pressure may make caregivers feel heavier burden.

Based on the identified significant factors, targeted interventions can be developed to alleviate AE caregiver burden, with detailed implementation feasibility and cultural considerations in the Chinese context: (a) *Anxiety Management*: Implement routine psychological screening and brief intervention programs for caregivers during the patient’s hospitalization, which can be integrated into the pre-discharge education workflow of Chinese neurology wards. Peer support groups led by experienced AE caregivers are also culturally adapted to the Chinese family care model, as they provide relatable emotional support and practical experience sharing; (b) *Caregiver Preparation*: Develop structured, easy-to-understand pre-discharge training programs covering AE disease knowledge, basic care skills, emergency response, and stress coping strategies. These programs can be delivered via a combination of offline lectures and online video tutorials, which are highly accessible for caregivers in China; (c) *Experience Sharing*: Establish a formal peer mentorship program in collaboration with patient organizations, where experienced AE caregivers provide one-on-one guidance for novice caregivers. This model is well-suited to the Chinese cultural context, as it leverages the strong sense of community among patient families; (d) *Economic Support*: Advocate for the inclusion of AE-related medical and rehabilitation expenses into the critical illness insurance coverage in China, and connect low-income caregivers to the medical assistance programs provided by local civil affairs departments. We also suggest flexible work arrangements for caregivers, which are supported by China’s current labor protection policies. These interventions, tailored to address the key drivers of burden identified in this study, can enhance caregivers’ wellbeing and ultimately improve the quality of care for AE patients. Future research should explore the long-term effectiveness of these targeted strategies and examine potential interactions between the identified factors.

## Limitations

This study has several intrinsic limitations that need to be acknowledged. The study cannot establish causal relationships between the identified factors and caregiver burden, only associations. Longitudinal studies are needed to confirm the causal pathways. We used convenience sampling from a single tertiary hospital in Shanghai, which may limit the generalizability of the results to primary care settings, rural areas of China, or international populations with different healthcare systems. We did not assess some factors that may influence caregiver burden, including caregiver social support, family function, patient neuropsychiatric symptoms, and long-term prognosis. These unmeasured factors may have additional effects on the results. Additionally, patient functional status (mRS and BI) was measured at admission, while caregiver burden was measured at discharge. Although admission severity is a key determinant of initial caregiver stress, patient condition may change during hospitalization. Future longitudinal studies should measure patient functional status at both admission and discharge, as well as during long-term follow-up, to better understand the dynamic relationship between patient disease trajectory and caregiver burden over time.

## Conclusion

In conclusion, primary caregivers of AE patients experience significant care burden, which is independently influenced by four key factors: caregivers’ anxiety level, care preparedness, previous care experience, and ability to afford medical expenses. These findings highlight that caregiver-centric characteristics, rather than patient-related factors, play a pivotal role in shaping the care burden of this population. Future research should focus on exploring the specific mechanisms underlying the interactions between these influential factors and conducting longitudinal studies to examine the dynamic changes in burden over the disease trajectory. In clinical practice, targeted interventions are warranted to alleviate caregiver burden: strengthening psychological support to manage anxiety, developing structured training programs to enhance care preparedness, establishing peer mentorship initiatives to facilitate knowledge and experience sharing among caregivers, and expanding economic support measures to reduce the pressure of medical expenses. By implementing these tailored strategies, we can effectively reduce the care burden of AE patients’ caregivers, improve their quality of life, and ultimately create a more supportive care environment that indirectly promotes the rehabilitation outcomes of AE patients.

## Data Availability

The raw data supporting the conclusions of this article will be made available by the authors, without undue reservation.

## References

[1] Aboseif A Kim NN Bou G Nathoo N Guo Y Pique J . Meningitis as an attack phenotype of myelin oligodendrocyte glycoprotein antibody–associated disease. JAMA Neurol. (2025) 82:871–3. doi: 10.1001/jamaneurol.2025.1774, 40522676 PMC12340653

[2] Gu Y Liu X Dong T Yang Q Bao X Zhao D . Anti-N-methyl-D-aspartate receptor type autoimmune encephalitis with severe pneumonia: a case report. World J Emerg Med. (2024) 15:142–6. doi: 10.5847/wjem.j.1920-8642.2024.024, 38476532 PMC10925533

[3] De Bruijn MAAM Leypoldt F Dalmau J Lee S-T Honnorat J Clardy SL . Autoimmune encephalitis. Nat Rev Dis Primers. (2025) 11:65. doi: 10.1038/s41572-025-00650-1, 40935848

[4] Yang R Ge F Jiang J Wang Y Zhang W. Disease characteristics, treatment, and prognosis of chinese patients with autoimmune encephalitis: a retrospective study. J Sichuan Univ Med Sci. (2022) 53:142–8. doi: 10.12182/20220160206, 35048615 PMC10408844

[5] Sun Y Li G Liu X Zhao X Ren J Ren G . Cerebral glucose hypometabolism and hypoperfusion of cingulate gyrus: an imaging biomarker of autoimmune encephalitis with psychiatric symptoms. J Neurol. (2024) 271:1247–55. doi: 10.1007/s00415-023-12051-z, 37945763 PMC10896782

[6] Seifert-Held T Eberhard K Lechner C Macher S Hegen H Moser T . Functional recovery in autoimmune encephalitis: a prospective observational study. Front Immunol. (2021) 12:641106. doi: 10.3389/fimmu.2021.641106, 34093529 PMC8175889

[7] Liu F Zhang B Huang T Wang B Wang C Hao M . Influential factors, treatment and prognosis of autoimmune encephalitis patients with poor response to short-term first-line treatment. Front Neurol. (2022) 13:861988. doi: 10.3389/fneur.2022.861988, 35493830 PMC9046540

[8] Bekdemi̇R A İLhan N. Predictors of caregiver burden in caregivers of bedridden patients. J Nurs Res. (2019) 27:e24. doi: 10.1097/jnr.0000000000000297, 30431539 PMC6553964

[9] Ahn S LaNoue M Su H Moale AC Scheunemann LP Kiehl AL . Post–intensive care syndrome and caregiver burden: a post hoc analysis of a randomized clinical trial. JAMA Netw Open. (2025) 8:e253443. doi: 10.1001/jamanetworkopen.2025.3443, 40198074 PMC11979734

[10] Argueta D Roberts S Brice K Wu-Chung L Lai V Paoletti-Hatcher J . Burden, grief, and depression in dementia spousal caregivers: the roles of inflammation and attachment security. Brain Behav Immun. (2024) 122:4–5. doi: 10.1016/j.bbi.2024.12.039

[11] Pillas D Klein A Gasalla T Avbersek A Thompson A Wright J . The burden of progressive supranuclear palsy on patients, caregivers, and healthcare systems by PSP phenotype: a cross-sectional study. Front Neurol. (2022) 13:821570. doi: 10.3389/fneur.2022.821570, 35865639 PMC9295700

[12] Zarit SH Reever KE Bach-Peterson J. Relatives of the impaired elderly: correlates of feelings of burden. Gerontologist. (1980) 20:649–55. doi: 10.1093/geront/20.6.649, 7203086

[13] Lu L Wang L Yang X Feng Q. Zarit caregiver burden interview: development, reliability and validity of the Chinese version. Psychiatry Clin Neurosci. (2009) 63:730–4. doi: 10.1111/j.1440-1819.2009.02019.x, 19781014

[14] Archbold PG Stewart BJ Greenlick MR Harvath T. Mutuality and preparedness as predictors of caregiver role strain. Res Nurs Health. (1990) 13:375–84. doi: 10.1002/nur.4770130605, 2270302

[15] Liu Y Wang M Dong XF. Reliability and validity of Chinese version of the caregiver preparedness scale in caregivers of stroke survivors. Chin J Pract Nurs. (2016) 32:1045–8. doi: 10.3760/cma.j.issn.1672-7088.2016.14.002

[16] Zung WWK. A rating instrument for anxiety disorders. Psychosomatics. (1971) 12:371–9. doi: 10.1016/S0033-3182(71)71479-0, 5172928

[17] Quinn TJ Dawson J Walters MR Lees KR. Reliability of the modified Rankin scale: a systematic review. Stroke. (2009) 40:3393–5. doi: 10.1161/STROKEAHA.109.557256, 19679846

[18] Haggag H Hodgson C. Clinimetrics: modified Rankin scale (mRS). J Physiother. (2022) 68:281. doi: 10.1016/j.jphys.2022.05.017, 35715375

[19] Fang Y Jiang Y Ma L Chen H Li Z Luo F . Effects of social support provided by disabled older adults to others on their own depressive symptoms: a moderated mediation model. Psychol Res Behav Manag. (2024) 17:3049–65. doi: 10.2147/PRBM.S468342, 39192967 PMC11348934

[20] Ariffin NHZ Yahya WNNW Hassan MR Mahadzir H. Factors affecting caregiver burden in older stroke survivors, a Malaysian study. Neurol Asia. (2025) 30:75–85. doi: 10.54029/2025cmz

[21] Zheng J Li R Wang Y Liu H Sun Q. The level and influencing factors of caregiver burden among primary caregivers of stroke patients in old age. Chin J Pract Nurs. (2016) 32:421–4.

[22] Alvarez Polo MT Sanchez Guarnizo RM Gómez Morales DF. Relationship between caregiver characteristics and the reported quality of life of people with mild and moderate dementia. Rev Cuid. (2025) 16:e4646. doi: 10.15649/cuidarte.4646, 40989542 PMC12453005

[23] Qian L. Analysis of burden of caregivers for Alzheimer disease patients and its influencing factors. Chin J Mod Nurs. (2017) 23:3227. doi: 10.3760/cma.j.issn.1674-2907.2017.25.009

[24] Springate BA Tremont G. Dimensions of caregiver burden in dementia: impact of demographic, mood, and care recipient variables. Am J Geriatr Psychiatry Off J Am Assoc Geriatr Psychiatry. (2014) 22:294–300. doi: 10.1016/j.jagp.2012.09.006, 23567422 PMC3723767

[25] Cejalvo E Martí-Vilar M Gisbert-Pérez J Badenes-Ribera L. Stress as a risk factor for informal caregiver burden. Health Care (Don Mills). (2025) 13:731. doi: 10.3390/healthcare13070731, 40218029 PMC11988590

[26] Ershad Ahmad Khan E Arifin WN Musa KI. Stroke survivors’ dependency level and informal caregivers’ quality of life in Kelantan, Malaysia: examining the mediating role of psychological distress. Cureus. (2024) 16:e64160. doi: 10.7759/cureus.64160, 39119401 PMC11309742

[27] Baig MO Khokhar MM Qureshi AP Ayan Z Batool M. Caregiver burden in family members of patients undergoing rehabilitation. Biosci Res. (2021) 18:3193–200. Available online at: https://www.isisn.org/BR18(4)2021/3193-3200-18(4)2021BR21-467.pdf

[28] Köther AK Alpers GW Büdenbender B Lenhart M Michel MS Kriegmair MC. Predicting decisional conflict: anxiety and depression in shared decision making. Patient Educ Couns. (2021) 104:1229–36. doi: 10.1016/j.pec.2020.10.037, 33248869

[29] Amin Abdelhalim DS Ahmed MM Hussein HA Sarhan MD Khalaf OO. A skill-based multimodal intervention for dementia caregivers: impact on burden and anxiety. Aging Clin Exp Res. (2025) 37:95. doi: 10.1007/s40520-025-02985-x, 40095192 PMC11914238

[30] McGuigan K Laurente G Christie A Carswell C Moran C Yaqoob MM . Effectiveness of interventions for informal caregivers of people with end-stage chronic illness: a systematic review. Syst Rev. (2024) 13:245. doi: 10.1186/s13643-024-02641-x, 39342397 PMC11438131

[31] Burke T Galvin M Pinto-Grau M Lonergan K Madden C Mays I . Caregivers of patients with amyotrophic lateral sclerosis: investigating quality of life, caregiver burden, service engagement, and patient survival. J Neurol. (2017) 264:898–904. doi: 10.1007/s00415-017-8448-5, 28280986

[32] Langford B Zhou Y Miyasaki JM. Multiple system atrophy caregivers’ experience: a mixed methods study. Can J Neurol Sci J Can Sci Neurol. (2023) 50:49–59. doi: 10.1017/cjn.2021.252, 34742360

[33] Tessitore A Marano P Modugno N Pontieri FE Tambasco N Canesi M . Caregiver burden and its related factors in advanced Parkinson’s disease: data from the PREDICT study. J Neurol. (2018) 265:1124–37. doi: 10.1007/s00415-018-8816-9, 29516169 PMC5937896

[34] Güner Y Çilingir D. Evaluation of caregiver burden of family members providing support for the care of patients undergoing brain surgery at the hospital. Florence Nightingale J Nurs. (2021) 29:167–75. doi: 10.5152/FNJN.2021.19207, 34263235 PMC8245025

[35] Uhm KE Jung H Woo MW Kwon HE Oh-Park M Lee BR . Influence of preparedness on caregiver burden, depression, and quality of life in caregivers of people with disabilities. Front Public Health. (2023) 11:1153588. doi: 10.3389/fpubh.2023.1153588, 37564425 PMC10409988

[36] X Z Z J Zou J Gao S W A Z Q. Dyadic transmission of depression in the elderly people with disabilities to caregiver burden: multiple mediating roles of caring ability and resilience. J Cent South Univ Sci Technol Med Sci. (2023) 48:1243–51. doi: 10.11817/j.issn.1672-7347.2023.220324, 37875365 PMC10930853

[37] Gillard PJ Ganapathy V Graham GD DiBonaventura MD Goren A Zorowitz R. Caregiver burden, productivity loss, and indirect costs associated with caring for patients with poststroke spasticity. Clin Interv Aging. (2015) 10:1793–802. doi: 10.2147/CIA.S91123, 26609225 PMC4644168

[38] Graus F Titulaer M Balu R. A clinical approach to diagnosis of autoimmune encephalitis. The Lancet Neurology. (2016) 15:391–404. 26906964 10.1016/S1474-4422(15)00401-9 PMC5066574

